# Conflicts over host manipulation between different parasites and pathogens: Investigating the ecological and medical consequences

**DOI:** 10.1002/bies.201600060

**Published:** 2016-08-11

**Authors:** Nina Hafer

**Affiliations:** ^1^ Department of Evolutionary EcologyMax‐Planck‐Institute for Evolutionary BiologyPlönGermany

**Keywords:** conflict of interest, host manipulation, multiple infections, parasite‐parasite interactions

## Abstract

When parasites have different interests in regard to how their host should behave this can result in a conflict over host manipulation, i.e. parasite induced changes in host behaviour that enhance parasite fitness. Such a conflict can result in the alteration, or even complete suppression, of one parasite's host manipulation. Many parasites, and probably also symbionts and commensals, have the ability to manipulate the behaviour of their host. Non‐manipulating parasites should also have an interest in host behaviour. Given the frequency of multiple parasite infections in nature, potential conflicts of interest over host behaviour and manipulation may be common. This review summarizes the evidence on how parasites can alter other parasite's host manipulation. Host manipulation can have important ecological and medical consequences. I speculate on how a conflict over host manipulation could alter these consequences and potentially offer a new avenue of research to ameliorate harmful consequences of host manipulation.

## Introduction: Host manipulation and multiple infections

To reproduce, parasites need to survive long enough to complete their life cycle. In some cases, they also need to move to a different habitat – be it a different host or the host's habitat. Unlike free living organisms, parasites rely on their host's behaviour and/or appearance to do so. Often this involves behaviours that go against the host's interests and might even be fatal for the host. To overcome this hurdle, some parasites have evolved host manipulation, i.e. the ability to alter the behaviour and/or appearance of their host in a manner that enhances their own fitness beyond the benefits they gain from exploitation. Therefore, the host may no longer be in full control of its own behaviour 1, 2.

Host manipulation occurs in a wide range of host and parasite taxa, and can take a large variety of forms (reviewed by 3, 4, 5, 6, 7, 8); furthermore, it can have important consequences for the ecosystem 1, 9, 10, 11, 12, 13. For example, host manipulation is likely to affect the energy flow in food webs. In the most obvious case, where a parasite enhances the predation susceptibility of its current host to its subsequent host, the trophic link between current and subsequent host is strengthened 1, 12. Humans too could be affected by host manipulation both as a final host of vector transmitted parasites (e.g. Malaria, 14, 15, 16) and as potentially accidental intermediate hosts (e.g. *Toxoplasma*, 17, 18, 19, 20).

Most studies on host manipulation have focused on single parasite species or even individuals. By contrast, hosts in nature are often infected by multiple parasites (e.g. 21, 22, 23, 24). Such a host is a conglomeration of various organisms, all of which might have different optima for host behaviour. If any of the parasites alter the host, such as by manipulating host behaviour and/or appearance, the host environment and possibly the fitness of any co‐infecting organism will be altered 1, 25, 26. This can result in a conflict between co‐infecting parasites if their interests collide 25, 27. In this essay, I review the current state of knowledge on conflicts over host manipulation and their outcomes with regard to host bebaviour, and speculate on its potential impacts, for example on ecology and medicine.

## Host manipulation is very diverse

### Host manipulation can enhance parasite transmission at the expense of host survival

Enhanced risk of an intermediate host to be preyed upon by a subsequent host (predation enhancement) probably represents the prime example of host manipulation. Complex life cycle parasites that require trophic transmission often enhance the predation susceptibility of their current host to ensure that they will reach their next host. Such parasites manipulate their hosts in a diversity of ways. Hosts might become more conspicuous to potential predators, develop physical impairments or lose their natural fear of (certain) predators (reviewed by 3, 4, 7, 8, 28). Naturally, successful predation enhancement is fatal for the host. Parasites that require a different habitat for reproduction and/or dispersal induce their hosts to move to such a habitat. The host usually dies in the process 29, 30, 31. Vector transmitted parasites depend on their vector, usually an insect, to disperse between hosts, plants or animals, when their vector feeds upon those hosts. To ensure transmission, parasites change the feeding behaviour of their vector and enhance encounter rates between vectors and hosts by altering host preferences of their vectors, and attractiveness of infected hosts 14, 15, 16, 32, 33, 34, 35, 36. Contact transmitted parasites, would also benefit from manipulating host behaviour to enhance encounter rates between infected and not yet infected hosts, though evidence for this is less clear than in the other cases (reviewed by 5).

### Host manipulation can enhance host survival

Parasites can also manipulate in a manner that – often temporarily – protects their host. Many parasites need to spend some time inside their intermediate host or vector before they are ready to be transmitted to the next host. During this time they temporarily reduce the mortality of their host (predation suppression) 14, 37, 38, 39, 40, 41. Parasitoids can manipulate their hosts to guard them even after emergence to avoid predation (body guard manipulation) 42, 43, 44. Such a host may eventually recover, albeit with severe reduction in fitness 45.

### Host manipulation is not restricted to ‘classic’ parasites

Even symbionts or commensals might benefit from hosts behaving in a certain manner. Hence, as in parasites, host manipulation (i.e. behavioural alteration induced by and beneficial to the symbiont/commensal) could have evolved 46. Vertically transmitted organisms can often benefit from altered sexual behaviour causing a number of changes in host mating and reproduction to ensure transmission (reviewed by 46, 47). There is increasing evidence that host microbiota, too, can alter host behaviour. The microbiome can secrete neuroactive components 48, and seems to influence eating behaviour and feeding preferences, presumably for reasons related to its own nutrition 26, 49, 50. Microbiota could further alter mood, personality traits and social preferences (reviewed by 26, 49, 50). Even cancer is suspected as potentially manipulating host behaviour for improved growth, and access to suitable nutrients by altering appetite and sleeping patterns 51. Not only “classic” parasites should have an interest in host behaviour, especially if it is altered by a manipulating parasite 25, 27.

## Different host manipulation can result in a conflict of interests

### Conflict over host manipulation can occur if parasites with contradictory interests infect the same host

**Figure 1:**
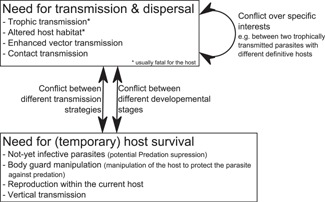
The diversity of host manipulation leading to potential conflicts over host manipulation. Parasites manipulate their hosts in a manner that increases their transmission and dispersal, often resulting in the host's death, or (temporarily) ensures their survival. Such mutually exclusive aims create the potential for conflict over host manipulation if different parasites infect the same host.

If two manipulating parasites with contradictory aims and different host manipulation co‐occur within the same host, there is potential for a conflict over host manipulation. However, there is no reason to assume that a non‐manipulating parasite has no interest in host behaviour. On the contrary, its fitness might simply be highest in a normally behaving host. If such a parasite and a manipulating parasite share a host, they too could be in a conflict over host manipulation 27. A conflict over host manipulation has been studied almost exclusively using at least one trophically transmitted parasite (Table 1). Whether such a conflict over host manipulation will occur depends on the specific evolutionary interests of each of the parasites involved. For example, it can occur between parasites with different transmission strategies (e.g. trophic transmission vs. reproduction within the current host or vertical transmission), different specific interests (e.g. different subsequent hosts) and different developmental stages (i.e. infective vs. not yet infective) (Fig. 1). This last potential conflict can occur between parasites of the same species if they represent different developmental stages.

**Table 1:** Overview and outcome of potential conflicts over host manipulation between parasites

Host	Parasite 1				Parasite 2				Outcome of the conflict	Ref.
	Species	Aim	Manipulation^a^	Proposed mechanisms	Species	Aim	Manipulation^a^	Proposed mechanisms		
**Conflict between parasites with different definitive hosts**										
*Gammaruspulex* (amphipod)^N^	*Pomphorhynchuslaevis* (Acanthocephala)	TT to fish	PE: strongly reduced photophobia	Immune system, neuromodulation, serotonin 101	*Polymorphus minutus* (Acanthocephala)	TT to birds	PE: Increased vertical distribution, slightly reduced photophobia	Immune system, neuromodulation, serotonin 101	Intermediate vertical distribution, *P. laevis* dominates reaction to light	55
*Paracalliopefluviatili* (amphipod)^N^	*Acanthocephalusgalaxii* (Acanthocephala)	TT to fish	PE: reduced photophobia	Immune system, neuromodulation, serotonin 101	*Microphallus* sp. (Trematoda)	TT to birds	None		No clear differences between singly infected hosts	87
*Batillariaattramentaria* (mollusc)^N^	*Cercariabatillariae* (Trematoda)	Transmission of cercariae to fish	Habitat change to much lower depths	Unknown	Renicolidae (Trematoda)	Transmission of cercariae to snails	Habitat change to slightly lower depths	Unknown	Intermediate habitat change	56
*Rattusnorvegicus* (mamal)^E^	*Toxoplasma gondii* (Protozoa)	TT to felines	Partially altered activity	Brain damage, dopamine, testosterone 102	*Toxocaracanis* (Nematoda)	TT to canids	Partially decreased activity	Debilitation 103	No clear differences between singly infected hosts	58
*Mus musculus* (mamal)^E^	*Toxoplasma gondii* (Protozoa)	TT to felines	Partially altered activity	Brain damage, dopamine, testosterone 102	*Toxocaracanis* (Nematoda)	TT to canids	None, but see 104	Debilitation 103	No clear differences between singly infected hosts	57
**Conflict between different transmission strategies (i.e. trophic transmission vs. growth and reproduction on/ in the current host)**										
*Gammarusinsensibilis* (amphipod)^NE^	*Microphalluspapillorobustus* (Trematoda)	TT to birds	PE: more risk prone reaction to disturbance	Immune system, neuromodulation, serotonin 101	*Gammarinemagammari* (Nematoda)	Ectoparasitic lifestyle	None		Hosts naturally infected with more *G. gammari* less manipulated, but not experimentally inducible	60
*Gammarusroeseli* (amphipod)^N^	*P. minutus* (Acanthocephala)	TT to birds	PE: strongly increased vertical distribution	Immune system, neuromodulation, serotonin 101	*Dictyocoela*sp (Microsporidae)	Vertical transmission	Slightly increased vertical distribution	Unknown	Co*‐*infections resemble*Dictyocoela*sp. infected hosts	59
**Intraspecific conflict between different developmental stages (i.e. trophic transmission vs. growth and development in the current host)**										
*Caecidotea intermedius* (isopod)^N^	*Acanthocephalusdirus* (infective) (Acanthocephala),	TT to fish	PE: colour change	Carotenoid based colouration of the parasite 105	*A. dirus* (not‐yet infective)	Growth and development	Slight colour change	Carotenoid based colouration of the parasite 105	Co‐infections resemble hosts with infective parasites	61
*G. pulex* ^NE^	*P. laevis* (infective) (Acanthocephala),	TT to fish	PE: reduced photophobia	Immune system, neuromodulation, serotonin 101	*P. laevis* (not‐yet infective)	Growth and development	None 73		Infective stages dominates, trend for slight effect of the not‐yet infective stage	63
*Macrocyclops albidus* (copepod)^E^	*Schistocephalus solidus* (infective) (Cestoda),	TT to fish	None, but see 39, 65, 106	Unknown	*S. solidus* (not‐yet infective)	Growth and development	PS: Reduced activity	Unknown	Infective stage dominates, no effect of one or multiple not yet infective stages	64
*M. albidus* ^E^	*Camalanus lacustris* (infective) (Nematoda),	TT to fish	PE: slightly increased activity, slightly reduced recovery time	Unknown	*C. lacustris* (not‐yet infective)	Growth and development	PS: Reduced activity, increased recovery time	Unknown	Infective stage dominates, no effect of one not yet infective stages	40
*Gasterosteus aculeates* (fish)^E^	*S. solidus* (infective) (Cestoda),	TT to birds	PE: increased risk taking	Brain monoamines 107, energy drain 76, 108	*S. solidus* (not‐yet infective)	Growth and development	None		No clear differences between singly infected hosts, mixed infections increase risk taking beyond infective parasites	76
**Interspecific conflict between different developmental stages (i.e. trophic transmission vs. growth and development in the current host)**										
*M. albidus* ^E^	*C. lacustris* (infective) (Nematoda),	TT to fish	PE: slightly increased activity, slightly reduced recovery time	Unknown	*S. solidus* (not‐yet infective)	Growth and development	PS: Reduced activity and increased recovery time	Unknown	Infective stage dominates, no effect of one not yet infective stages	40
*M. albidus* ^E^	*S. solidus* (infective) (Cestoda),	TT to fish	None, but see 39, 65, 106	Unknown	*C. lacustris* (not‐yet infective)	Growth and development	PS: Reduced activity and increased recovery time	Unknown	Both parasites affect host behaviour	40

N, Natural infections; E, Experimental infections; PE, Predation enhancement; PS, Predation suppression; TT, Trophic transmission.

^a^ Only host manipulation observed by the study investigating the conflict. For many host‐parasite systems host manipulation of the same or other traits has been shown by other studies.

The existence of a conflict over host manipulation should manifest itself in differences in the behaviour of singly infected hosts. Host manipulation can be rather unspecific. For example, some trophically transmitted parasites alter host behaviour in a manner likely to enhance predation not only by the correct consecutive hosts, but by non‐host predators alike (e.g. 52, 53, 54). If two such unspecific manipulators share the same host, a conflict over host manipulation between them is unlikely even if they have different subsequent hosts 27. From a practical point of view, a conflict should manifest itself in significantly different behaviours between singly‐infected hosts. Matters are further complicated if two parasites with contradictory aims manipulate the host differently, but in the same direction. In such a case, some functional experiments would be necessary to test whether transmission to each host is maximised by an optimal level of host manipulation or whether one parasite is simply unable to induce the maximal level of host manipulation 25.

### Conflicts can result in compromise, persistence or suppression of host manipulation

#### Conflict over different specific interests can result in a compromise

If two manipulating parasites infect the same intermediate host but need different subsequent hosts, there is potential for conflict between them. To study this conflict, Cézilly et al. 55 compared the behaviour of wild caught gammarids (small shrimp‐like crustaceans) that harboured a fish‐ or a bird‐infecting acanthocephalan parasite. Gammarids harbouring the fish‐infecting parasite show strongly reduced photophobia, whilst those harbouring the bird‐infecting parasite show only slightly reduced photophobia and occur higher up in the water column. These changes are assumed to facilitate trophic transmission to their respective hosts. Hosts with both parasites occur in intermediate water depths, whilst their photophobia is similarly reduced as that of hosts infected by the fish parasite. The fish parasite was also the stronger manipulator in single infections. In another experiment investigating the combined effect of two trematode species on mud snails, snails naturally infected with either of the two parasite species show a different spatial distribution from each other and from uninfected snails 56. These trematodes are transmitted to their subsequent hosts, either fish or snails, when the current snail host releases infective stages (cercariae) into the water. The particular position on the shore of infected snails is hypothesised to facilitate the release of cercariae in close proximity to their respective hosts. The distribution of co‐infected snails is intermediate 56. Since these studies both used naturally infected hosts, some caution is warranted when interpreting their results. Two studies have investigated the joint influence of the dog infecting nematode *Toxocara canis* and the cat infecting protozoon *Toxoplasma gondii* on various behaviours of their common intermediate hosts, mice 57 and rats 58, using experimental infections. Doubly infected rats slightly resemble *T. canis* rats in their behaviour 58. However, singly infected animals behave similarly to each other 57, 58, not illustrating any clear conflict between the parasites. These studies indicate that two parasites with conflicting interests could both have an effect on host behaviour leading to host behaviour that unites traits of hosts that are singly infected by either parasite. Nevertheless, the stronger manipulator might have an advantage 55.

#### Conflict between different transmission strategies can result in suppression

If parasites differ in their transmission strategies there is potential for a conflict over host manipulation. Such conflict will occur if, for example, a trophically transmitted manipulating parasite shares a host with a parasite that reproduces within this host or is vertically transmitted. Gammarids serve as intermediate hosts for the bird infecting acanthocephalan parasite *Polymorphus minutus*. Infected hosts occur higher in the water column. The microsporidian *Dictyocoela* sp. depends on the same gammarid for vertical transmission. Hosts infected only by the microsporidian parasite occur only slightly higher in the water column than their uninfected counterparts. Predation by the bird due to host manipulation would be fatal for the microsporidian. Hosts harbouring both the bird parasite and the microsporidian parasite occur in similar water column heights as hosts with the microsporidian parasite only 59. Similarly, gammarids harbouring a trophically transmitted trematode are less likely to show altered responses to a disturbance if they also harbour nematodes for which predation would be fatal. However, cure from, and reinfection with, the nematodes fails to cure or reintroduce this effect 60. This illustrates the need for studies using experimental infections to test the joint effect of parasites with different transmission strategies on host behaviour.

#### Experimental studies have investigated the outcome of conflicts between different developmental stages

##### The infective stage performs better in an intraspecific conflict

Many parasites need to spend some time inside their intermediate host to grow and develop before they are infective to the next host. During this time, successful host manipulation by another infective parasite would be fatal for the not yet infective parasite. This temporarily results in a similar scenario as a conflict between parasites with different transmission strategies; one parasite depends on the host, whilst the other one manipulates in a manner that, if successful, results in the host's death. Such a conflict can even occur between parasites of the same species. Isopods naturally infected by a manipulating acanthocephalan parasite have an altered colour pattern making them more conspicuous to bird predators, the parasite's subsequent host. After the parasite has reached infectivity, the alteration in the colour pattern becomes much more pronounced. In infections that combine the infective with the not yet infective parasite, colour patterns are as pronounced as in infections with only the infective parasite 61.

**Figure 2:**
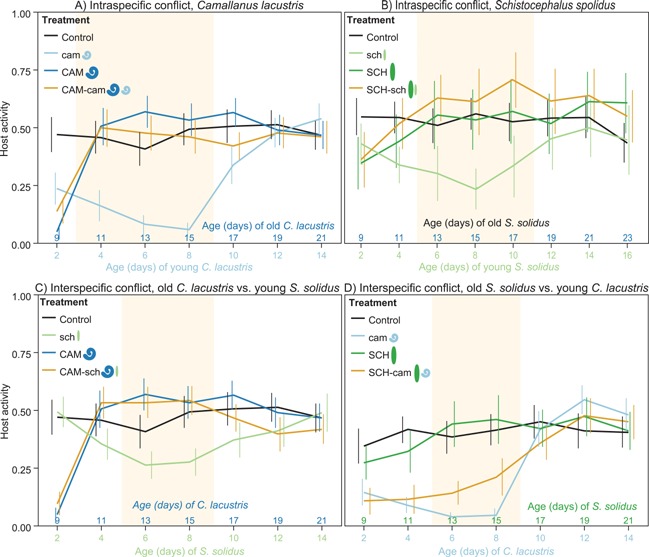
Outcome of a conflict over host manipulation between different developmental stages in the copepod *Macrocyclops albidus*. Host activity correlates positively with predation susceptibility by the subsequent host, a fish 65. **A**: Intraspecific conflict within *Camallanus lacustris*, **B**: intraspecific within *Schistocephalus solidus*, **C**: interspecific conflict between an old (infective) *C. lacustris* and a young (not yet infective) *S. solidus* **D**: interspecific conflict between an old (infective) *S. solidus* and a young (not yet infective) *C. lacustris*. Control: uninfected control copepods, cam: Copepods with a young *C. lacustris*, CAM: copepods with an old *C. lacustris*, CAM‐cam: copepods with an old plus a young *C. lacustris*, sch: copepods with a young *S. solidus*, SCH: copepods with an old *S. solidus*, SCH‐sch: copepods with an old plus a young *S. solidus*, CAM‐sch: copepods with an old *C. lacustris* plus a young *S. solidus*, SCH‐cam: copepods with an old *S. solidus* plus a young C*. lacustris*. Shaded areas indicate time during which a conflict over host manipulation occurs, that is, significant differences in behaviour in copepods infected by either parasite. Error bars indicate 95% CI. Error bars of control copepods have been omitted for easier readability (**A**, **C** and **D** after 40, **B** after 64).

A conflict between different developmental stages seems particularly attractive to study using experimental infections. Unlike studies on naturally infected hosts, these studies can establish cause and consequence between the altered behaviours observed in an infected host, and its infection status 25, 62. For example, Dianne et al. 63 tested a conflict between infective and not‐yet infective stages of an acanthocephalan parasite in its gammarid host. Infective parasites strongly reduce photophobia. Coinfection by a not yet infective stage might slightly reduce this host manipulation. In two similar studies, an infective cestode 64 or an infective nematode 40 manages to completely suppress any manipulation of its copepod host (ancient group of small aquatic crustaceans) by a not yet infective conspecific despite being the weaker manipulator when acting alone (Fig. 2A and B). In this system, the activity of the host is strongly reduced by not yet infective parasites 39, 64, hence, preventing premature predation 38. Once the parasite reaches infectivity, host activity increases to similar or slightly higher levels as in uninfected control copepods 39, 64 and predation is enhanced 65. The infective parasite seems to do better in an intraspecific conflict over host manipulation irrespectively of how strongly it manipulates when alone.

##### An interspecific conflict can resemble an intraspecific conflict

Within the same species or between closely related species, parasites are likely to use the same proximate mechanisms to manipulate. This should facilitate cross‐talk between them, and could facilitate modification of another parasite's host manipulation 25. For example, cross talk between parasites could facilitate suppression of host manipulation between different developmental stages of the same parasite species 40, 64. With that in mind, Hafer and Milinski 40 investigated the same conflict, but using two phylogenetically distinct parasite species, a cestode and a nematode (Fig. 2C and D). Akin to an intraspecific conflict, the infective nematode is able to completely suppress host manipulation by the not yet infective cestode. If an infective cestode and a not yet infective nematode share their host, both clearly influence host activity, resulting in intermediate host activity compared to singly infected hosts. This is consistent with the fact that the nematode also appears to be the stronger manipulator. This study shows that one parasite can suppress host manipulation by another parasite even between species. Such suppression might be modified by how strongly each parasite manipulates. Even distantly related parasites can use similar mechanisms to manipulate host behaviour 66, maybe explaining the striking resemblance of host manipulation between different parasites species and the fact that it can – at least partially – be suppressed by other, not closely related, parasites 40.

## What determines the outcome of conflict over host manipulation?

### Costs and benefits of a conflict, and encounter rates between parasites, should shape selection

#### Parasites face costs and benefits in a conflict

Theoretical models predict that costly predation enhancement is most likely to evolve when baseline transmission rates are low 67 and parasite mortality is high 67, 68. Energetic costs are usually assumed to restrict host manipulation 5, 11, 67, 68, 69 and similar costs should apply to its suppression, but neither has ever been measured directly. Conflicting host manipulation by co‐infecting parasites will reduce transmission rates (e.g. predation suppression) or increase mortality (e.g. increased (dead‐end) predation), and hence, should select for suppression of this host manipulation (Table 2). If the not yet infective parasite loses in a conflict between different developmental stages, its mortality through fatal premature transmission increases. For the infective parasite losing this conflict merely results in delayed transmission (Table 2). Nevertheless, in all studies to date the infective parasite seems mostly to prevail in its manipulation, and at least partially supress manipulation by a not yet infective parasite 40, 61, 63, 64. The evolution of predation suppression by not yet infective parasites is limited by a trade‐off with resource acquisition needed for parasite growth and development 68, and maintaining the host long enough and in sufficiently good condition to ensure later transmission. These restrictions should also apply to the evolution of the suppression of host manipulation, and might even be heightened by the presence of an additional parasite that drains energy and potentially harms the host.

**Table 2:** Costs and benefits of losing and winning a conflict over host manipulation and the observed outcomes of such conflicts

Conflict over	Parasite	Hypothetical costs of sabotage	Consequences of losing the conflict	Consequences of winning the conflict	Factors potentially favoring parasite in a conflict	Empirical outcomes of the conflict
Different definitive hosts	Either	Energetic costs	Death	Transmission		Intermediate host behaviour 55, 56 or one parasite persist in its host manipulation 55
Different transmission strategies	Trophically transmitted parasite	Energetic costs	Reducedtransmission	Transmission	Strength of host manipulation^c^	Suppression by the non‐trophically transmitted parasite in natural infections 59, 60, but not experimentally reproducible 60
Different transmission strategies	Non‐trophically transmitted parasite	Energetic costs, physiological harm to the host	Death^b^	Survival^b^	Priority^d^	See row above
Different developmental stages	Infective parasite	Energetic costs	Delayed transmission, competition, mate availibility^a^	Transmission at an optimal time point	Size, Priority	No 61/possibly very weak 63 suppression by the not yet infective parasite, complete^40^, ^64^ or partial^40^ suppression by the infective parasite
Different developmental stages	Not yet infective parasite	Energetic costs, physiological harm to the host	Death	Transmission, competition, mate availibility^a^		See row above

^a^ Only applies in an intraspecific conflict and if parasites are of opposite sexes or hermaphroditic. Benefits depend on the likelihood of encountering a mate in the definitive host and the costs of failing to do so.

^b^ Fitness consequences will depend strongly on how much of its potential reproduction a parasite has already realised prior to its host becoming infected by the manipulating parasite.

^c^ In case of a non‐manipulating co‐infecting parasite for which ‘normal’ host behaviour would be optimal.

^d^ In case of a vertically transmitted parasite.

In a conflict over different transmission strategies, e.g. between trophically transmitted and non‐trophically transmitted parasites, successful transmission of the trophically transmitted parasites is also fatal for the non‐trophically transmitted parasite (Table 2). Reproduction prior to manipulation, however, could reduce the loss of fitness. When evolving suppression to host manipulation, the same restrictions that apply to not‐yet infective parasites should apply to non‐trophically transmitted parasites because they also need to ensure that they gain enough nutrients, and the host is maintained long and well enough for them to reproduce. Nevertheless, correlational evidence suggests that such a parasite can successfully supress host manipulation by trophically transmitted parasites 59.

#### Do encounter rates between specific parasites shape the conflict between them?

Even when the benefits of suppression outweigh the costs, whether or not selection pressures will be high enough for suppression to evolve will largely depend on how likely it is that a conflict occurs 70. The probability of encountering any one specific parasite might sometimes be low, but any parasite should encounter some other parasite, commensal or symbiont with potentially conflicting interests with regards to host behaviour. Hence, parasites should benefit from manipulating their host in a manner that switches off any previous and prevents any successive manipulation by any other parasite. Can they do so 25? A fish acanthocephalan parasite mostly persists in altering phototaxis of its intermediate host both when encountering a bird‐infecting parasite 55 or a not yet infective conspecific 63. Similarly, an infective nematode is able to successfully supress host manipulation by both a not yet infective conspecific and a not yet infective cestode 40. However, infective cestodes that are able to supress host manipulation by not yet infective conspecifics 64 only partly succeed in supressing host manipulation by not yet infective nematodes 40. More stringent studies investigating the outcome of conflicts between one parasite and multiple other parasites will be necessary, but challenging. Understanding the mechanisms of suppression offers an additional avenue of research to gain further insights into these questions.

### Proximate factors could influence a conflict over host manipulation

#### The first parasite to infect a host might be at an advantage

Do parasites that infect their host first have an advantage when it comes to a conflict over host manipulation? If the first parasite altered the host irreversibly, it might become a different habitat, possibly one less suitable for and susceptible to host manipulation by further parasites 1, 25, 71. In a conflict between different developmental stages, the infective stage is the one that has been inside the host for longer. It is also the one that performs better if there is conflict over host manipulation 40, 61, 63, 64, even in a combination of parasites that might be rare in nature 40. In a conflict between different developmental stages, a potential effect of infection order could be confounded by age and size. Parasite size 72, 73, 74 and number (reviewed by 25, 27) can influence the strength of host manipulation. They could determine how much of a manipulative or a manipulation‐supressing component a parasite is able to produce. In an intraspecific conflict between different developmental stages of a cestode, however, parasite size does not seem to influence the outcome of a conflict over host manipulation. Rather multiple not yet infective parasites are also unable to resist suppression by an infective conspecific 64. Unfortunately, studies using experimental infections in which infections take place sequentially are limited to a conflict between different developmental stages 40, 63, 64.

#### The original host manipulation and its mechanism could shape the conflict

Not every parasite that encounters another parasite in its host will have evolved strategies to deal with the presence of this specific parasite. In such cases, the original host manipulation might be decisive of the outcome of the conflict between them. A parasite that manipulates more strongly could be expected also to have a stronger effect on host manipulation in a shared host. In some cases this does indeed seem to be the case, but not in others (Table 1). Parasites manipulate by neuromodulation, immunomodulation, encystment at certain sites, and energy drain (reviewed by 75). Unfortunately, few different scenarios for a conflict over host manipulation have been investigated, and little in the way of specifics are known to determine general patterns with regards to the effect these mechanisms will have on the outcomes of conflicts over host manipulation (Table 1). Some mechanisms of host manipulation could be particularly difficult to counteract. For example, the fright response of sticklebacks harbouring an infective and a not yet infective cestode (i.e. parasites that should be at a conflict over host manipulation) is unexpectedly, even more reduced than that of hosts harbouring the infective parasite only. This could be explained if the altered fright response occurs due to a side‐effect of enhanced energy drain, which should be enhanced in double compared to single infections 76. The not yet infective parasite might have no means to prevent energy drain. Similarly, host manipulation of one parasite could be influenced by side‐effects caused by other parasites. For example, inducing certain behaviours such as enhanced activity in a severely sick host might be difficult 77. Thus, we need a better understanding of the mechanisms underlying host manipulation before we can understand how parasites interact on a proximate level when it comes to host manipulation, and how this might influence conflicts over host manipulation.

## Conflict over host manipulation could have far reaching consequences

### Conflict over host manipulation could modify the ecological consequences of host manipulation

#### Infected and uninfected hosts differ in their ecology

From an ecological perspective, a manipulated host is not the same as a non‐manipulated host. It retains some traits from the uninfected host, but other traits may be altered by host manipulation 1, 25, 71 and it can occupy a different ecological niche from an uninfected host 13, 78. Hence, host manipulation can result in two distinct phenotypes, i.e. infected and manipulated versus uninfected and not manipulated, each phenotype potentially occupying distinct ecological niches 1, 71. Thereby, competition between infected and uninfected hosts could be reduced 12. If multiple manipulating and/or suppressing parasites infect the same host population, the number of distinct phenotypes should increase, potentially restoring some overlap between them.

Manipulating parasites can also have effects that go beyond their current host. By altering its role in the food web, manipulating parasites change or even create energy flow through food webs 1, 12, 78, thereby, potentially altering food web structure and stability 1. This is especially obvious in trophically transmitted parasites that enhance predation susceptibility to facilitate transmission to a subsequent host. As a side‐effect, trophically transmitted parasites can also alter predation by non‐suitable dead‐end predators 53, 54, 79, 80. Additionally, parasites with different transmission strategies can affect food webs: examples are parasites that alter their host's habitat. For instance, some hairworms induce their terrestrial insect host to seek out water in which the host dies and becomes available as an otherwise unattainable food source (reviewed by 12). Manipulation or suppression by additional parasites should alter the food web further or reduce some effects of the original host manipulation 13.

Changes in trophic interactions by host manipulation will also affect interactions between invasive and native species 81, and thereby, shape the outcome of biological invasions. Depending on the circumstances it could slow down or speed up invasions 71, 82. Other parasites could further alter the effect of host manipulation on biological invasions depending on each parasite's interest. Invasive species can also bring their parasites with them, some of them potential manipulators. Some of these parasites could establish in their host's new habitat. How will such invasive parasites interact with the native parasites whose interests with regards to host manipulation might collide? Native and invasive parasite will not have coevolved, but even parasites that might not co‐occur very often can alter each other's host manipulation 40. Host manipulation clearly has ecological consequences. How these could be altered by other parasites requires further study.

#### Can a conflict over host manipulation alter infection patterns?

Host manipulation could influence infection patterns if parasites respond to the evolutionary pressures imposed by the presence of other manipulating parasites, for example, by avoiding wrongly manipulated hosts. However, studies looking at associations between parasites in naturally infected hosts have mostly failed to find any negative association between hosts with conflicting interests when it comes to host manipulation (59, 83, 84, 85, 86, 87, but see also 88). Interestingly, positive associations between parasites with similar interests seem to occur more frequently (reviewed by 25), but this could also be caused directly by the parallel life cycles 89. Clearly, there is need for experimental studies on whether host manipulation can affect infection patterns.

### Can hosts benefit from a conflict over host manipulation?

#### Suppression can restore host behaviour, but at what cost to the host?

If one parasite suppresses host manipulation by another parasite, this could benefit the host by restoring its original behaviour. However, this suppression could cause physiological damage, limiting its usefulness to the host. Parasites should limit this damage to accommodate their future need for that host. Vertically transmitted parasites, in particular, should avoid harming their host, because their reproduction directly depends on the host's reproduction, and therefore, their suppression should benefit the host. However, even in many complex life cycle parasites, multiply infected hosts show no increase in mortality 90 and can even be in better condition than singly infected hosts 91, 92.

#### Host manipulation and its suppression might be fine‐tuned by co‐evolution

Not all host manipulation might be detrimental for the host. Some hosts and parasites have co‐evolved potentially creating a balance of host manipulation, and hosts trying to counter it. If such a parasite were lost – or its host manipulation suppressed by another parasite – this could then lead to suboptimal host behaviour from the host's perspective 93. The same should be true for any co‐evolved three‐way interaction whereby a host has co‐evolved with two manipulating parasites keeping each other at bay. In this case, it would be the loss of one parasite that would expose the original host manipulation with its negative impacts on the host.

#### Can a conflict over host manipulation help to alleviate the medical and economic impacts of host manipulation?

Many diseases of economic and medical importance, such as Malaria 14, 15, 16, several other vector transmitted diseases (reviewed by 36, 71), and various pathogens of economically important crops 33, 34, 36, rely on vector transmission, and manipulate hosts and vectors to ensure transmission. Host manipulation itself can also affect humans and domestic animals (reviewed by 71). *Diplostomum* trematodes, for example, might increase predation by birds in fish farms 71 and *T. gondii* seems to be associated with changes in human personality traits up to severe psychological disorders 17, 18, 19, 20. Even our gut microbiota could change our behaviour facilitating obesity 50.

Do parasites (or symbionts or commensals) exist that suppress the adverse effect of such parasites? If so, they could be used to tackle harmful manipulation. Symbionts and vertically transmitted parasites can protect their hosts from more harmful parasites (reviewed by 85, 94, 95). *Wolbachia,* for example, is a prime candidate in fighting vector transmitted diseases because it has been shown to interfere with the transmission of some viruses that can causes diseases such as Dengue, Chikungunya, Yellow Fever, West Nile, as well as the infectivity of the malaria‐causing protozoan *Plasmodium* 96, 97. Antagonistic parasites are also increasingly used to fight pathogens in natural populations (reviewed by 77). Interactions between humans and their microbiota can provide some protection against diseases such as malaria 98, 99, 100. Do such interactions also affect host manipulation? Investigating the effect of other parasites and microbes on host manipulation by such parasites of medical or economic interest could help shed light on this question. The approach of using actual parasites would of course be limited by their adverse effects, at least when dealing with humans and domestic animals. However, a better understanding of the mechanisms that such parasites use to supress other parasite's host manipulation might aid in the development of drugs to achieve the suppression of unwanted host manipulation.

## Conclusions

If there is a conflict over host manipulation, host manipulation by one parasite can be altered up to its complete suppression by other, co‐infecting parasites from either the same or a different species. Unfortunately, only a limited number of studies have investigated a conflict over host manipulation, and they are limited to very few, nearly exclusively aquatic, host taxa. Even fewer studies have been done using experimental infections. Nearly all of these studies have focused on conflicts involving at least one trophically transmitted parasite. Host manipulation, of course, is much more diverse, and evidence is accumulating that it is not limited to parasites. Even in a seemingly healthy host, host behaviour may not be under the sole control of the host 1, 2, 25, 26, 48. In addition, organisms that do not alter the behaviour of their hosts might still benefit most from hosts behaving in a certain manner, in this case ‘normal’. Hence, a conflict over host manipulation could be much more frequent than the number of empirical studies would indicate.

Many potential impacts of host manipulation on ecology and medicine exist. A conflict over host manipulation and the potentially resulting suppression of one parasite's host manipulation by another can alter the impacts of host manipulation. These alterations will have consequences that go well beyond the immediate host behaviour and could potentially help us to deal with negative impacts of host manipulation. Nevertheless, the possible impacts of a conflict over host manipulation and its outcomes have received even less attention than the conflict over host manipulation itself.

The authors have declared no conflict of interest.
